# Supplementary material for: Understanding How School-Based Interventions Can Tackle LGBTQ+ Youth Mental Health Inequality: A Realist Approach
Source: Int J Environ Res Public Health. 2023 Feb 28;20(5):4274. doi: 10.3390/ijerph20054274 (PMC10001812; doi:10.3390/ijerph20054274)
Supplement: Supplementary file 1 [file ijerph-20-04274-s001.zip › ijerph-2045358-supplementary.pdf]

CLASS
CREATING LGBTQ+ AFFIRMING SCHOOL ENVIRONMENTS

Young People WhatsApp interview schedule

Interview preamble

Participants will be sent via WhatsApp or email the following documents at least one week before the interview:

- Participant information infographic and information video
- Consent form

At least one day before the interview, participants will be sent the following infographic:

HOW TO PREPARE FOR YOUR WHATSAPP INTERVIEW

BE DATA AWARE

Will you have stable wifi or wifi you will be using to take part in the interview? Or do you have good signal to be able to use your data to take part in the WhatsApp interview?

We will invite you to delete our chat as soon as the interview is over, but you may wish to check in advance that it is not backed up anywhere else. Find out if you have backups enabled on your WhatsApp chat here: WhatsApp>Settings>Chats>ChatBackup

BE COMFY

Pick a place that's comfortable and private, where you will feel happy to WhatsApp chat for up to an hour.

PART 1 – INTRODUCTION

First, interviewer to check that the participant has received and had a chance to look over consent form, information infographic, and information video. Are there any questions related to that?

Interviewer to send to participant the following pre amble information:

- **What the interview is for**

We have developed some ideas about interventions that can support anti homophobic, transphobic, biphobic bullying and would like to hear what you think about these suggestions and how we can develop these further.

- **What to expect today**

We have found seven main types of support that are in place at schools to help LGBTQ+ young people. I would first like to talk to you about any strategies that you may have experienced yourself and whether you have found them helpful or not. I would like to ask you four main questions to understand why you thought they helped or not.

The interview should take between 30 minutes to 1 hour.

We really want to understand *why* things work, so I am going to ask you for lots of detail. Any information that you provide is really helpful – there are no right or wrong answers.

- **Opting out**

If you decide that you no longer want to take part at any point, we can stop the interview. I can also delete the recording of the interview if you would like me to.

- **Anonymity and Confidentiality**

Everything you say in the interview will be kept confidential. This means that what you tell me will not be shared with other people. The only time things you tell me will not be kept confidential is if you tell me that you or another person is at serious risk of harm. This is called safeguarding (this is to keep you safe).

The text from this interview will be taken and anonymized so all of your personal details are removed. I will delete our WhatsApp chat as soon as I have created an anonymized version of our conversation.

Your contributions will be anonymized, this means that your name and any specific locations, school names and other names will be removed and you will not be named in anything written about the study.

- **After the interview**

After the interview, you can ask for us to delete your interview contributions for up to two weeks after today.

After the interview, if you want to talk about anything we have raised today then you can discuss it with a trusted adult. At this school, we have identified XXX as a teacher that would be open to talking to you about anything you wanted to discuss.

- **Any questions?**

- **Consent form completion**

- **Check willingness to continue**

Supplementary Material

<u>MAIN BODY OF INTERVIEW</u>

Send section images as below:

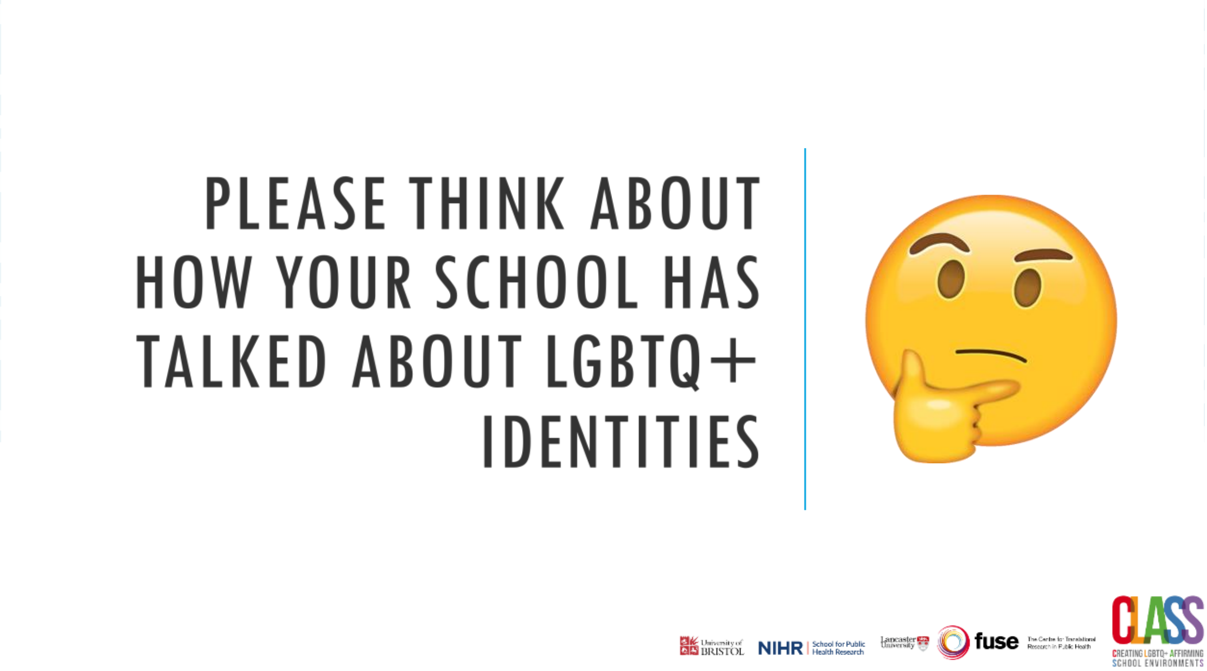

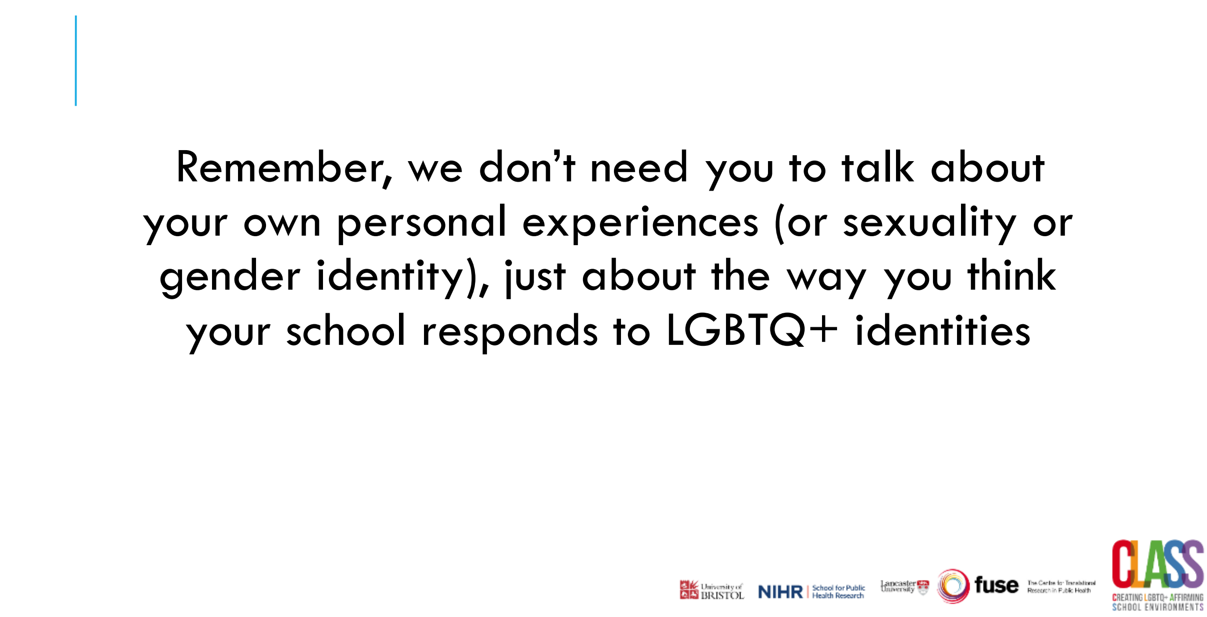

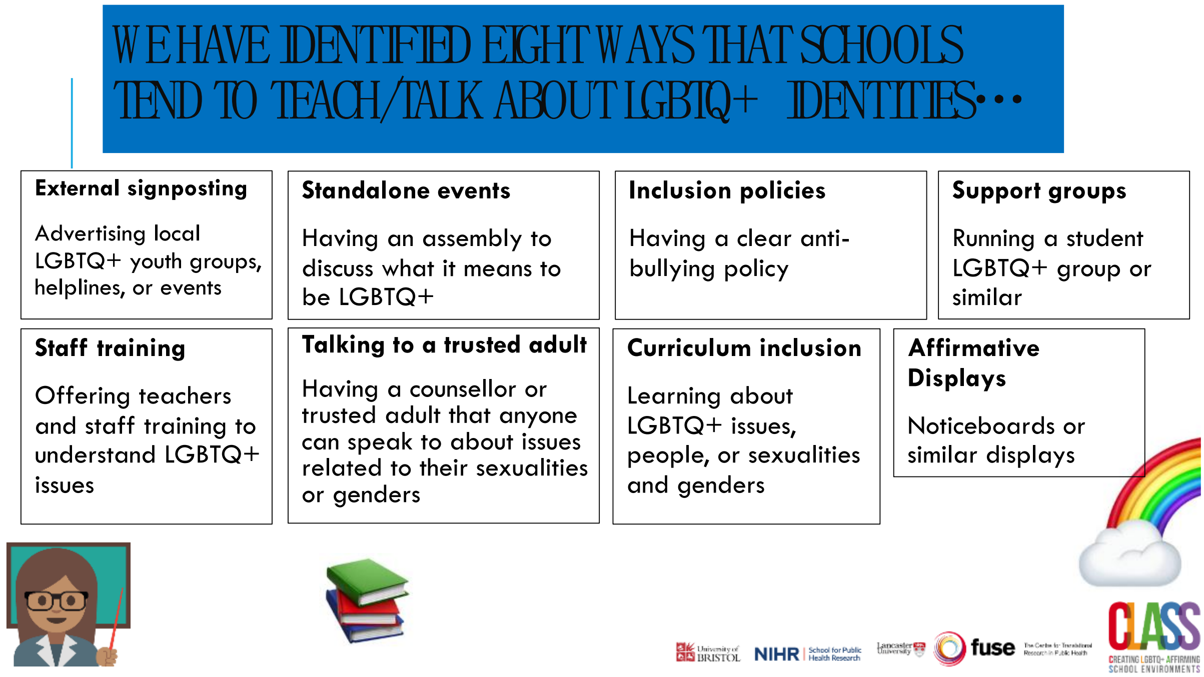

SEND QUESTION IMAGES ONE BY ONE:

Question 1:

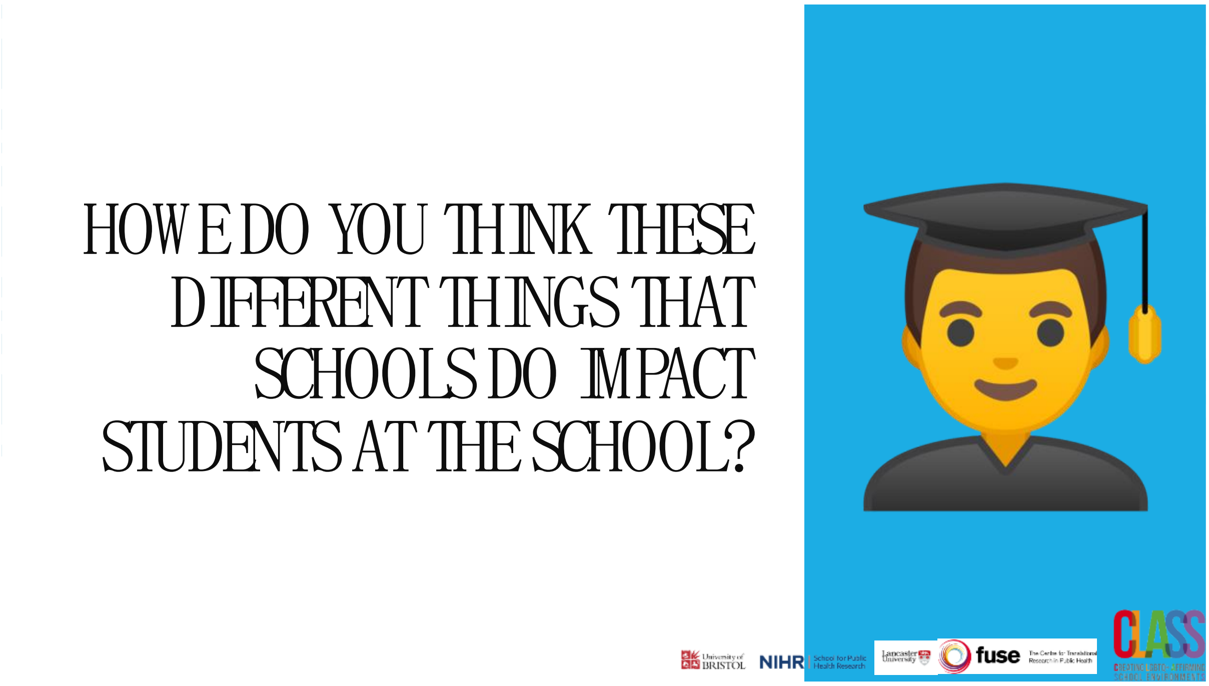

Prompts: School climate, culture, environment;
Relationships and interactions;
Behaviours & actions;
Self, thoughts, beliefs;
Affect, feeling, emotions

Question 2:

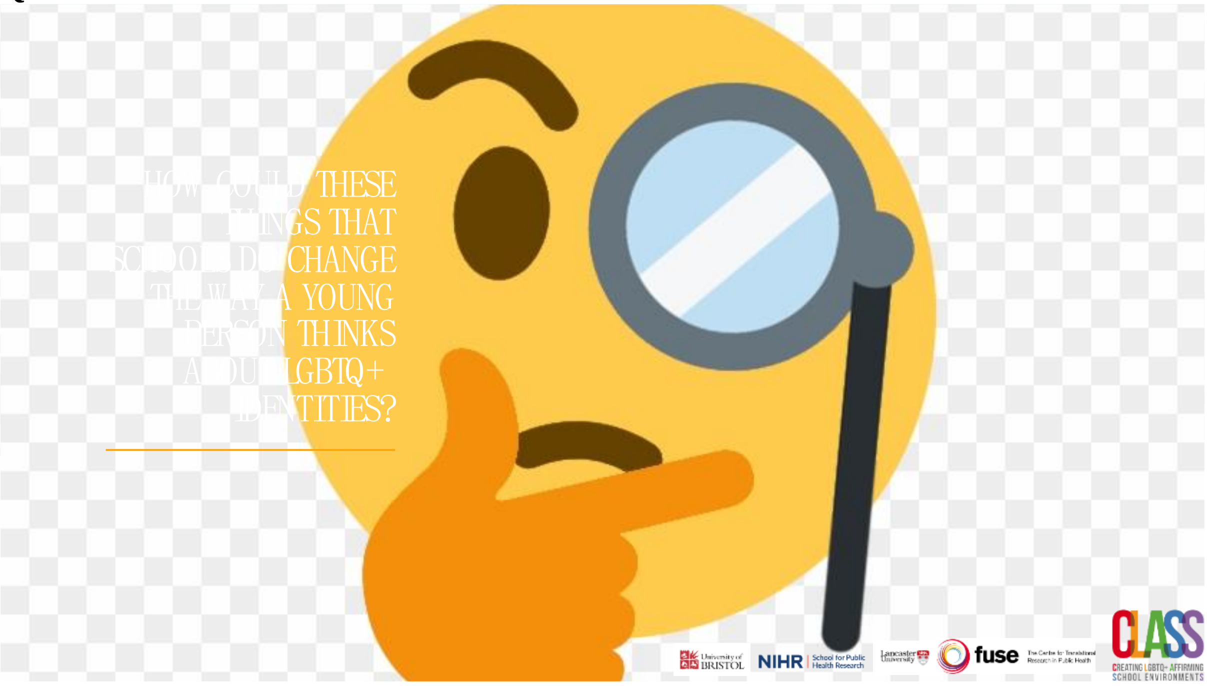

Prompts:

- *Affirmation (I am good);*
- *Agency (I can do);*
- *Advocacy (I can make better);*
- *Belonging (I am included);*
- *Connectedness (I am like you);*
- *Coping (I am positive);*
- *Recognition (I count);*
- *Safety (I feel no fear);*
- *Usualising (I am accepted)*

Question 3:

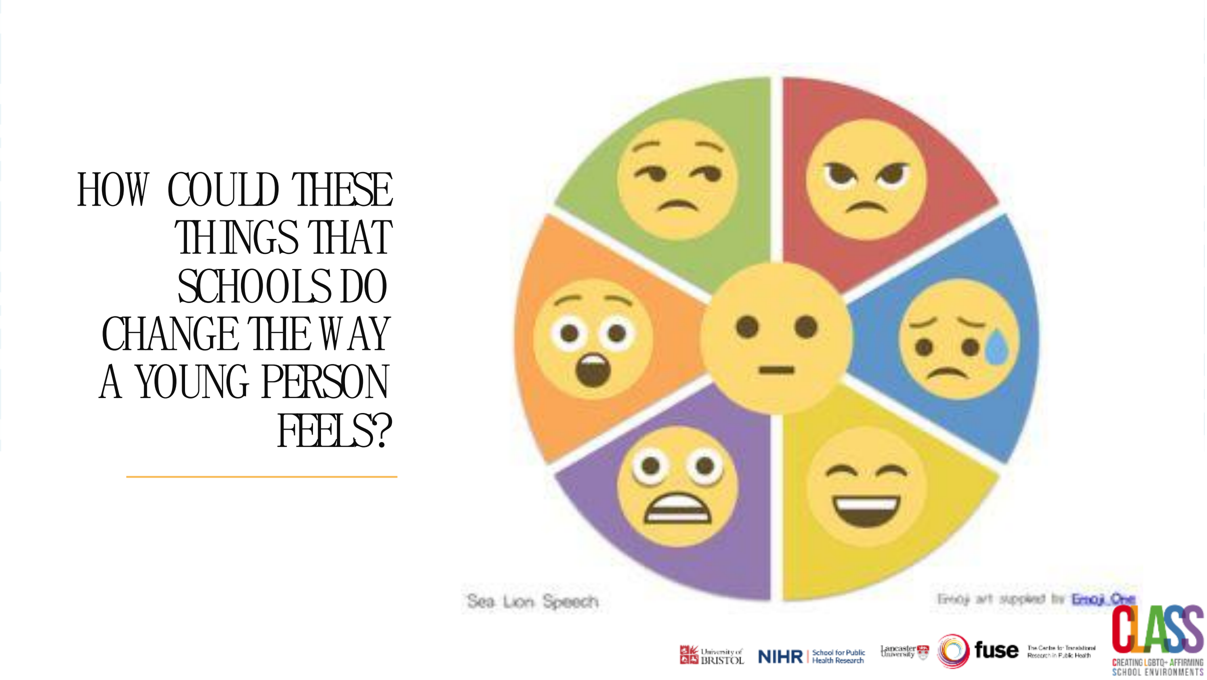

Prompts: Does it reduce any negative emotions, does it increase any positive emotions?

Question 4:

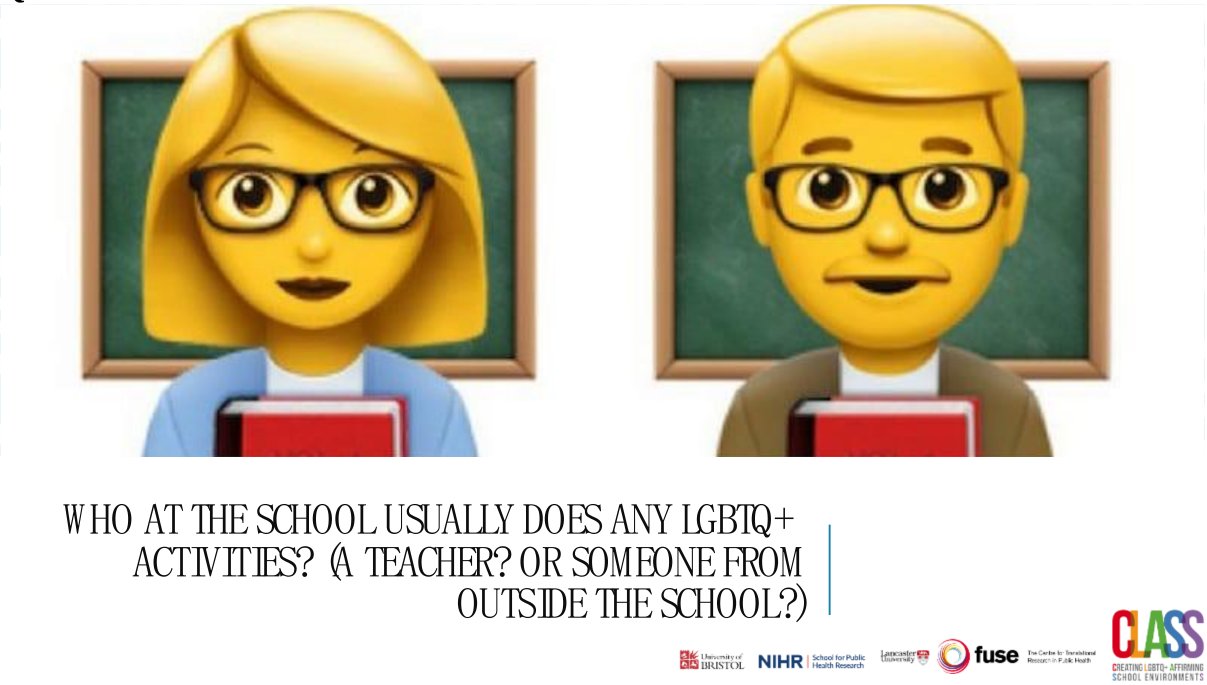

(*Prompts: qualities of deliverer, training, experience)*

WHO ATTENDS THE DELIVERY OF LGBTQ+ RELATED EVENTS?

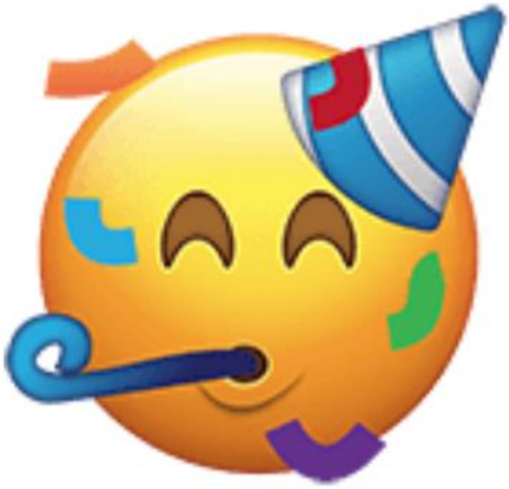

(*Prompts: Displayed in prominent areas, LGBT+ only or do heterosexual young people also attend*?)

HOW ARE THE LGBTQ+ EVENTS TALKED ABOUT?

For example, are they celebratory? Or do they focus on the risks related to gender and sexuality?

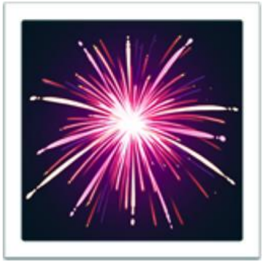

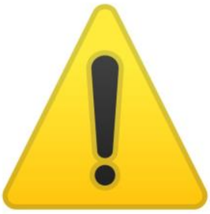

(*Prompts: E.g. positioning LGBT+ as 'at-risk'? Celebrating diversity*)

PART 3 – INTERVIEW EXIT

Interviewer to do the following:

- Demographics – send link to an online form they can fill in to list their demographic information
- Thank participant
- Welfare check
- Remind of site specific trusted adult
- Send resource slide (as below)

Support contacts

YOUR GP can help you. Ring the surgery for an out of hours telephone number

SAMARITANS – 116 123
- Call free from any phone **24 hour a day**. Support for those experiencing distress, despair and/or suicidal feelings.
- Email: jo@samaritans.org

NHS 111 – 111
- Call free 24 hours a day – non emergency line
- Call 999 in the event of an emergency

Places that could help a young LGBQ person you may know:

CHILDINE – 0800 1111

Free, **24-hour** helpline for children and young people aged up to 19 years old in trouble or danger. If the lines are busy, please keep trying.

1-2-1 counsellor chat available at: https://www.childline.org.uk/get-support/1-2-1- counsellor-chat/

LGBT Foundation – 0345 3 30 30 30 or 0161 235 8035
- Helpline run by an experienced LGBT charity available 9am until 9pm Monday to Friday, and 10am until 6pm Saturday.
- Email: helpline@lgf.org.uk, website: http://www.lgf.org.uk/

ALLSORTS – 01273 72 12 11
- **Allsorts is charity which supports young people under 26 who are lesbian, gay, bisexual, trans or unsure (LGBTQU) of their sexual orientation and/or gender identity.** http://www.allsortsyouth.org.uk/

GALOP - 0800 999 5428
- National LBGT domestic advice helpline.
- Email: help@galop.org.uk

Switchboard – 0300 330 0630
- LGBT+ helpline available 10am to 10pm everyday
- Email: chris@switchboard.lgbt

Albert Kennedy Trust **- Newcastle** 0191 281 0099
- Call for support for LGBT 16-25 year olds who are made homeless or living in a hostile environment.

- Allow time for post interview questions and thoughts until participant leaves the interaction
